# Peutz-Jeghers Type Polyp of the Appendix with Review of Literature

**DOI:** 10.1155/2019/7584070

**Published:** 2019-07-25

**Authors:** Jolanta Jedrzkiewicz, Keith Quencer, Anna P. Matynia, Ellen Morrow, Maria Pletneva, Gonzalo Barraza

**Affiliations:** ^1^Department of Pathology, University of Utah, 1950 Circle of Hope, Salt Lake City, UT 84112, USA; ^2^Department of Radiology, University of Utah, 1950 Circle of Hope, Salt Lake City, UT 84112, USA; ^3^Department of Pathology, University of Utah and ARUP Laboratories, 500 Chipeta Way, Salt Lake City, UT 84108, USA; ^4^Department of Surgery, University of Utah, 50 N Medical Dr., Salt Lake City, UT 84132, USA; ^5^George E. Wahlen Department of Veterans Affairs Medical Center, Department of Pathology, 500 Foothill Dr, Salt Lake City, UT 84148, USA

## Abstract

Hamartomatous polyps of Peutz-Jeghers type are strongly associated with Peutz-Jeghers polyposis syndrome and are predominantly encountered in the small intestine. Sporadic cases are uncommonly reported. We report a case of a polyp identified incidentally in the appendix of a patient undergoing diagnostic imaging due to a history of hepatitis C infection. Histopathologic evaluation after appendectomy showed a polyp with bands of muscularis mucosae bundles with arborizing architecture and variable amounts of inspissated mucin, morphologically indistinguishable from Peutz-Jeghers type hamartomatous polyp. A family or personal history of abdominal cancers was not reported by the patient, suggesting a sporadic occurrence. Next generation sequencing revealed only two pathogenic low-level* STK11* mutations, presumed to be somatic. In conclusion, this is an unusual case of a sporadic Peutz-Jeghers type polyp occurring in the appendix.

## 1. Introduction

Appendiceal dilatation of more than 6 mm detected on abdominal imaging can potentially trigger appendectomy due to concern for presence of infection or malignancy [1]. The preoperative differential diagnosis is broad, including inflammatory conditions, benign tumors, and malignant neoplasms. The final diagnosis is often unknown until the results of the histopathologic analysis of the resected tissue become available.

The most common tumors involving the appendix are mucinous tumors, such as low grade appendiceal mucinous neoplasm (LAMN) and mucinous carcinomas, adenomas, serrated polyps, goblet cell tumors, neuroendocrine tumors, and colonic type carcinomas [2]. Hamartomatous polyps can also occur in the appendix but are very rare, mostly described in case reports [3]. Unlike adenomas, which are largely sporadic, hamartomatous polyps of Peutz-Jeghers type are mostly associated with polyposis syndromes such as Peutz-Jeghers polyposis, juvenile polyposis, or Cowden syndrome, and this diagnosis may trigger clinical evaluation for cancer predisposition in the affected patient.

Sporadic Peutz-Jeghers type polyps are considered very uncommon and are mainly documented in case reports and small case series [4, 5]. Additionally, the risk for cancer predisposition in these cases is currently uncertain, as there is limited clinical follow-up in the available literature. In one of the available studies aiming to evaluate sporadic Peutz-Jeghers type polyps, there were only 8 out of 102 Peutz-Jeghers polyps identified over a period of 22 years that could have potentially been sporadic [4]. Most of these polyps occurred in middle aged to older patients and about half of them developed a malignancy, suggesting a possible link between even the sporadic Peutz-Jeghers type polyps and cancer predisposition [4].

## 2. Case Presentation

**Figure 1:**
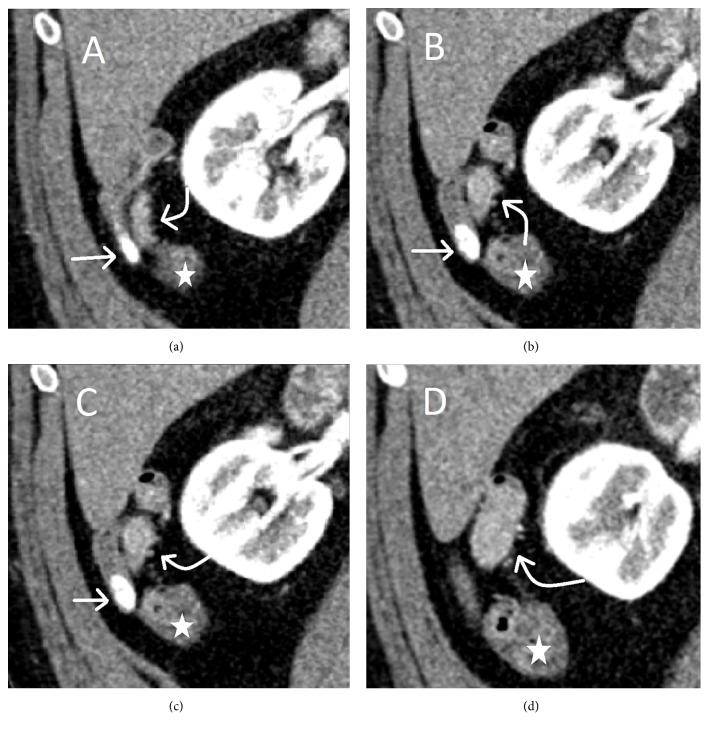
Coronal image from a contrast enhanced CT shows the appendiceal mass. At the widest portion (D) the appendix measured 16 mm in diameter. Cecum (star). Appendicolith at the tip of appendix (straight arrow). Enhancing appendiceal mass concerning mucinous tumor of the appendix (curved arrow).

A 60-year-old male with a prior history of alcoholism, tachycardia, hypertension, schizophrenia, and hepatitis C infection underwent regular surveillance protocol for hepatocellular carcinoma. Imaging revealed an incidental dilatation of appendix, which slowly increased in size over a 10 year period. More recent images revealed a dilatation of the midportion of the appendix measuring 16 mm in widest dimension, concerning for a mucinous appendiceal tumor (Figure 1) and an appendicolith in the distal appendix. There was no surrounding inflammation identified to suggest rupture radiographically. Subsequent colonoscopy showed a single hyperplastic polyp and normal appearing appendiceal orifice. The patient's family and personal history was negative for abdominal cancers, hamartomatous polyps, or mucocutaneous pigmentations.

The patient was referred for a surgical consult due to increase in size of this appendiceal lesion and progressive luminal dilatation. Ultimately, an appendectomy was pursued due to concern for the presence of a mucinous tumor of the appendix. During the procedure, the appendix appeared dilated without signs of perforation or carcinomatosis. The appendix was resected without complication and was submitted intact for histopathologic evaluation.

**Figure 2:**
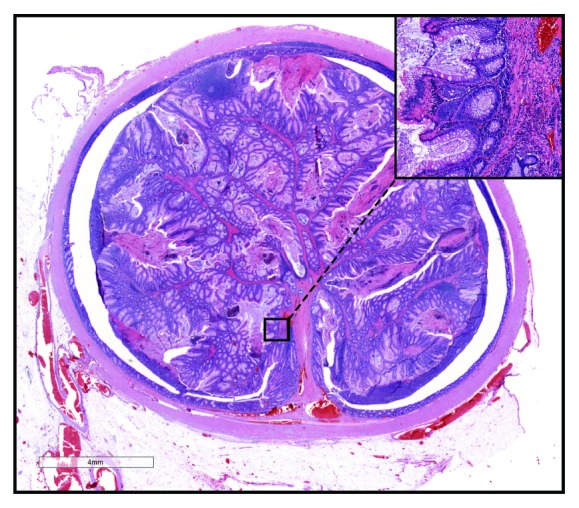
H and E sections of appendiceal lesion at low magnification (1X) show pedunculated polyp with arborizing bands of fibromuscular tissue (20X), distending appendiceal lumen.

Gross examination revealed a 1.4 cm well-circumscribed pale brown intraluminal mass in the mid portion of a 7.5 x 2.5 x 2.2 cm appendix, 4 cm from the resection margin. Microscopically, the mass appeared to be a pedunculated polyp supported by broad bands of arborizing fibromuscular bands at low power, lined by colonic type epithelium without dysplasia (Figure 2). Altogether, the findings were in keeping with a Peutz-Jeghers type hamartomatous polyp, which markedly distended the appendiceal lumen. There was no evidence of adenomatous change, low grade mucinous neoplasia, or malignancy identified in any of the examined tissue sections.

**Figure 3:**
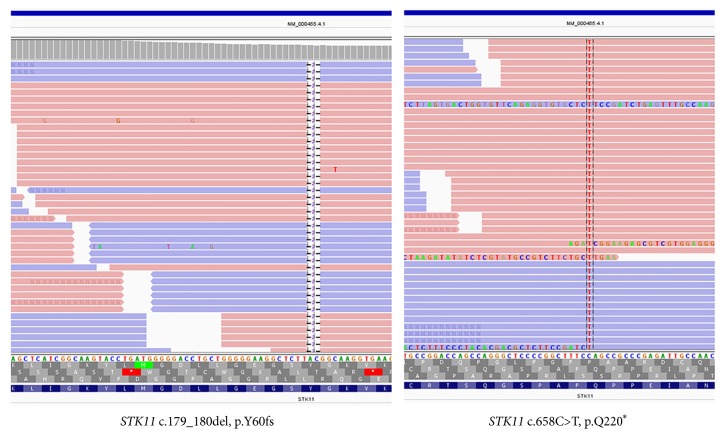
Visualization of two* STK11* mutations in Integrative Genomics Viewer (IGV).

The resected tissue was evaluated for pertinent mutations via a clinically validated solid tumor mutation panel by next generation sequencing (NGS) assessing mutational hotspot regions in 44 genes including* STK11* (exons 1, 4, 5, 6, and 8; NM_000455.4) and* PTEN *(exons 1-9; NM_000314.6). Genomic DNA was extracted from FFPE tissue followed by hybridization capture to enrich for the regions of interest and next generation sequencing. Two low-level mutations (presumed somatic) were detected in* STK11* gene: a frame-shift mutation in exon 1 (c.179_180del, p.Y60fs) at variant allele frequency (VAF) of 1.8% and a nonsense mutation in exons 5 (c.658C>T, p.Q220*∗*) at VAF of 2.7%. Even though both of these mutations are slightly below the established limit of detection (LOD) of 5% VAF of this assay, the high quality scores and bidirectional and staggered reads (Figure 3) support that these calls represent true mutations. No other variants were detected in any of the other genes included in the panel.

## 3. Discussion

Peutz-Jeghers polyps are diagnosed histologically based on the presence of broad bands of arborizing muscularis mucosae smooth muscle bundles, described as resembling a “Christmas tree” at low magnification, with variable amounts of inspissated pink mucin. Typically, the polyp is lined by benign epithelium without adenomatous change. The differential diagnosis includes adenomas, juvenile polyps, serrated polyps, inflammatory polyps, and mucosal prolapse. In the current case there was no adenomatous change to suggest a villous adenoma. Juvenile polyps and inflammatory polyps typically have more pronounced inflammatory components and dilatation of crypts. Features of mucosal prolapse include fibromuscular hyperplasia of the lamina propria extending towards the luminal aspect of the mucosa as well as distorted diamond shaped glands in the deeper aspect and congestion of dilated capillaries, and these features were not seen in the current case. The observed morphologic findings were all in keeping with a Peutz-Jeghers type polyp.

This case is uncommon because the Peutz-Jeghers type polyp presented in the appendix of a patient without clinical or familial history suggestive of a polyposis syndrome; therefore both the site and patient demographics were unusual. There are only rare documented reports of appendiceal Peutz-Jeghers polyps [3, 6–11]. Appendiceal Peutz-Jeghers polyps may present clinically with intussusception, while others occur incidentally [9]. To our knowledge this is the first report of a case presenting with dilatation of the appendix on imaging mimicking a mucinous appendiceal neoplasm.

Classic Peutz-Jeghers polyps occur predominantly in the small intestine, followed by the stomach and colon. There is a strong association with Peutz-Jeghers polyposis syndrome, which is a rare autosomal dominant condition, characterized by hamartomatous polyps in the gastrointestinal tract, mucocutaneous pigmentation, and a predisposition to a variety of neoplasms including carcinomas of the pancreas, colon, stomach, small bowel, breast, ovaries, and testes [12]. A thorough review of the clinical and family history in the current case failed to reveal typical lesions associated with Peutz-Jeghers syndrome, suggesting sporadic occurrence. Additionally, solid tumor NGS panel performed in this case showed only two pathogenic low-level* STK11* mutations. These mutations are presumed to be somatic based on low variant allele frequencies; however, their germline origin cannot be completely excluded as the assay performed is not designed to distinguish between somatic and germline variants. The exact role of these mutations in the pathogenesis of the polyp is uncertain. Additionally, the performed panel is designed to detect only single nucleotide variants (SNVs) and insertion and/or deletions (Indels) and only in the exons captured. Gross (e.g., exon-level) deletions (known to cause a subset of Peutz-Jeghers syndrome cases) would not be detected by this panel and their presence in this patient cannot be completely excluded. Although these results do not completely rule out the syndromic origin of the polyp, with the combination of the negative family and patient's history they make it much more unlikely.

In conclusion, we report a very unusual case of likely sporadic Peutz-Jeghers type polyp, which presented with a dilated appendix and was radiographically concerning mucinous tumor of appendix.
